# Analysis of 13-valent pneumococcal polysaccharide conjugate vaccine among children born in Hangzhou from 2017 to 2021

**DOI:** 10.3389/fpubh.2023.1184059

**Published:** 2023-05-30

**Authors:** Xinren Che, Qinghua Chen, Yan Liu, Lintao Gu, Zhaojun Lu, Wenwen Gu, Jun Wang, Wei Jiang, Jian Du, Xiaoping Zhang, Yuyang Xu, Xuechao Zhang, Jing Wang, Qixin Xie, Yingying Yang

**Affiliations:** ^1^Department of Expanded Program on Immunization, Hangzhou Center for Disease Control and Prevention, Hangzhou, Zhejiang, China; ^2^Department of Expanded Program on Immunization, Linping Center for Disease Control and Prevention, Hangzhou, Zhejiang, China

**Keywords:** 13-valent pneumococcal polysaccharide conjugate vaccine (PCV13), all-course vaccination rate, first vaccination rate, Hangzhou, children

## Abstract

**Background:**

13-valent pneumococcal polysaccharide conjugate vaccine (PCV13) has been introduced in Hangzhou since 2017, whereas its current immunization state in children is not clear. Therefore, this study aims to describe the PCV13 vaccination distribution among children born in Hangzhou from 2017 to 2021 to provide data for reducing vaccination differences among different populations.

**Methods:**

Descriptive epidemiology was used for data analysis and PCV13 vaccination related information of children was collected from children vaccination management system of Zhejiang Province (ZJCVMS).

**Results:**

Among the 649,949 children born in Hangzhou from 2017 to 2021, 169,230 were vaccinated with an average full course vaccination rate of 26.0%. The full course vaccination rates in 5 years were different (*P* = 0.000) with an increasing trend (*P*_fortrend_ < 0.01). The first dose vaccination rates were different in 5 years (*P* = 0.000) with an increasing trend (*P*_fortrend_ < 0.01). The distribution of age when first dose PCV13 was administered varied, most people at 2 months and least people at 5 months. The full course vaccination rate varied by areas, highest in central urban areas and lowest in remote areas respectively (all *P*-value < 0.05). Overall, the full course vaccination rate of PCV13 was higher in the registered residence population than the non-registered residence population, which was 136,693 (31.4%) and 32,537 (15.1%) respectively (*P* = 0.000). The full course vaccination rates were the same between men and women (*P* = 0.502), which was 87,844 for men (26.0%) and 81,386 for women (26.1%).

**Conclusion:**

Although the number of people who received PCV13 full course vaccination and received the first dose vaccination showed yearly increasing trends in Hangzhou, the full course vaccination rate for the whole population was relatively low. In addition, the PCV13 vaccination rates also differed by geography and household registration status. Measures such as expanding vaccination publicity or including national immunization should be taken to increase vaccination rates and reduce the differences in vaccination among groups with different characteristics.

## 1. Introduction

Pneumococcal disease (PD) is an infectious disease caused by *streptococcus pneumoniae* (Spn). According to the infection sites, PD can be divided into invasive pneumococcal disease (IPD) and non-invasive pneumococcal diseases (NIPD). Studies have shown that Spn can cause different types of infections, mainly IPD including meningitis, bacteremia and bacteremic pneumonia (1, 2). Some rare IPD infection types, including pericarditis (3), endocarditis (4), Austrian syndrome (5), necrotizing fasciitis (6), Hemolytic uremic syndrome (7), and Hemophagocytic syndrome (8), have been gradually emphasized in clinical settings. NIPD mainly includes Acute otitis media (AOM), sinusitis and non-bacteremic pneumonia.

PD is one of the most serious public health problems in the world. According to the research published in 2018, there are still about 294,000 children under 5 years old who died of PD in the world, and the incidence rate and mortality in developing countries and regions are higher than those in developed countries and regions. Plus, the vast majority of deaths occur in Africa and Asia (9). Worldwide, countries with top 10 highest number of under-5-year-old children PD cases are all in Africa and Asia, accounting for 66% of the global total number. It is noteworthy that China, with the second largest number of PD cases, accounted for 12% of the global total number (1). Spn is also an important cause of morbidity and mortality among infants and the older adults in China (10–15). PD has a serious economic burden such as hospital expenses, nursing expenses, and visiting expenses (16–20).

In addition, some studies have shown that Spn is resistant to commonly used antibiotics, such as penicillin, macrolides, cephalosporins and sulfonamides (21, 22). Epidemiological studies in China have shown that the problem of pediatric Spn resistance is increasingly serious (23, 24). Fewer and fewer antibiotics can be used clinically to treat IPD. Some studies also showed that drug-resistant Spn was less likely to be isolated with the introduction of pneumococcal vaccine on a large scale. The incidence of diseases caused by penicillin non-susceptible strains in children under 2 years old decreased by 81% from 70.3 per 100,000 to 13.1 per 100,000 (25).

Pneumococcal conjugate vaccine is the most economical (26) and effective (27, 28) measure to prevent pneumococcal related diseases under the circumstance of high economic burden and emergence of large number of drug-resistant Spn strains. After the introduction of PCV13 in the United States, the incidence of pneumococcal disease and untyped empyema decreased (29), and the incidence of non-antibiotic-sensitive IPDs decreased in multiple age groups (30). It is recommended by the WHO vaccine preventable disease classification guideline that PD and malaria are supposed to be the highly prioritized vaccine-prevented disease (31). A study of pneumonia incidence and PCV13 vaccination in children born in Ningbo, Zhejiang Province during 2017–2018 showed that the risk of pneumonia in those who had completed at least 3 doses of PCV13 vaccination was 0.582 times higher than that in those who had not received PCV13 vaccine. The vaccine protection rate was 41.80%. Markov model analysis in the above study showed that when PCV13 was included in herd immunity, the immediate medical cost was reduced by 28%, the number of averted infections increased by 186%, the number of averted deaths increased by 24 times, and the incremental cost-effectiveness ratio was reduced by nearly 85% (32).

By 2022, there are 198 vaccination clinics in Hangzhou, all of which are public. These clinics were responsible for the vaccination of Hangzhou's programmed and non-programmed vaccines. There are also a few private vaccination clinics that provide non-immunization program vaccinations, but the number of vaccines administered is small. There are 11 kinds of immunization program vaccines and 29 kinds of non-immunization program vaccines in use in Hangzhou, among which pcv13 is voluntarily vaccinated by citizens at their own expense. The price of PCV13 vaccine is relatively high, with PCV13-CRM197 698 yuan/dose and PCV13-TT 598 yuan/dose. In 2022, the per capita disposable income of Hangzhou residents was 70,300 yuan, and the price of a single PCV13 vaccine accounted for about 1% of the per capita income of Hangzhou. PCV13 has been introduced in Hangzhou since September 2017. In order to understand the PCV13 vaccination situation of children under 5 years old in Hangzhou, Zhejiang Province, and to provide data support for the inclusion of PCV13 in the free vaccination strategy on regional vaccination differences and the full course vaccination status.

## 2. Methods

### 2.1. Study population

This study analyzed the current state of PCV13 vaccination of children under 5 years old through collecting vaccination related information from children vaccination management system of Zhejiang Province (ZJCVMS). Children born between January 1, 2017 and December 31, 2021 in ZJCVMS were included in the study. On June 30, 2022, the researcher counted the PVC13 vaccination data from “ZJCVMS” by month.

### 2.2. Basic information and definitions

According to the distance from the central city of Hangzhou, it can be divided into central urban area, near central urban area or remote area. There are 14 districts or counties within Hangzhou, 6 are classified as central urban areas (Shangcheng, Gongshu, Xihu, Binjiang, Qiantang, and Fengjingmingsheng), 5 are classified as near central urban area (Xiaoshan, Yuhang, Linping, Fuyang, and Linan), and the rest are classified as remote areas (Tonglu, Jiande, and Chunan). ZJCVMS is a computer software system used to store children's personal information and vaccination information in Zhejiang Province.

“Local household registration” is defined as children registered in Hangzhou City. “Non-local household registration” is defined as children registered outside Hangzhou. “Full course vaccination” refers to the completion of PCV13 immunization program, that is, children have administered the full doses of PCV13 at the age required by PCV13 immunization program.

### 2.3. PCV13 vaccination procedures

There were two types of PCV in Hangzhou from 2017 to 2021, namely PCV13-CRM197 and PCV13-TT. Children's parents choose the vaccine voluntarily and at their own expense according to the procedure.

PCV13-CRM197: 2, 4, 6 months of age for basic immunization (no younger than 6 weeks), one dose each, 12 to 15 months of age for enhanced immunization, one dose.

PCV13-TT: Infants aged 2 to 6 months (no younger than 6 weeks): A total of 4 doses were given. It is recommended that the first dose to be given at 2 months of age (at least 6 weeks of age), and 3 doses of basic immunization are given, with an interval of 2 months between the two doses. The fourth dose was given at 12 to 15 months of age. Infants aged 7 to 11 months: 2 doses of basic immunization were given, with at least 2 months apart; One booster dose (the third dose) was given after 12 months of age and at least 2 months apart from the second dose. Children aged 12 to 23 months: 2 doses, at least 2 months apart. Children aged 2 to 5 years: 1 dose.

### 2.4. Statistical analysis technique

SPSS 21.0 (IBM Corporation, New York, USA) software was used for data analysis. Descriptive epidemiology was used to describe demographics of children born in 2017 to 2021 and current state of PCV13 vaccination. Counting data was described by rate and composition ratio. Chi-square test, and Chi-square trend test were used for data analysis, with test level α = 0.05.

### 2.5. Ethical considerations

This study was determined to be exempt from ethical review by the Hangzhou CDC institutional review board. Data was safe when extracted from ZJCVMS and not linked to individual identification.

## 3. Result

### 3.1. Demographics

The total registered population of ZJCVMS from 2017 to 2021 were 649,949, which was 157,002, 137,493, 135,044, 115,719, and 104,691 respectively in each year. Among the total population in 5 years, men and women were 337832 and 312117, accounting for 52.1 and 47.9% respectively. The population of central urban areas, near central urban, the remote areas were 263,179, 330,991, and 55,779, respectively, children accounting for 40.49, 50.9, and 8.6%. A total of 849,081 vaccinations were completed for children born in 2017–2021 (Table 1).

**Table 1:** PCV13 inoculation in Hangzhou from 2017 to 2021.

			**No. of children**	**First dose**				**Second dose**			**Third dose**			**Forth dose**				**Total**
				**PCV13-CRM197**	**PCV13-TT**	**Total of the firstdose**	**Vaccination rates of the first dose(%)**	**PCV13-CRM197**	**PCV13-TT**	**Total of the seconddose**	**PCV13-CRM197**	**PCV13-TT**	**Total of the thirddose**	**PCV13-CRM197**	**PCV13-TT**	**Total of the forthdose**	**Vaccination rates of the forth dose (%)**	
Region	central urban areas	Shagncheng	75,020	30,526	4,673	35,199	46.9	29,884	3,017	32,901	29,344	1,875	31,219	24,654	788	25,442	33.9	124,761
		Gongshu	55,937	24,456	2,388	26,844	48.0	23,969	1,666	25,635	23,541	1,318	24,859	19,586	754	20,340	36.4	97,678
		Xihu	58,469	23,312	4,751	28,063	48.0	23,111	3,695	26,806	22,698	2,839	25,537	19,396	1,597	20,993	35.9	101,399
		Binjiang	31,364	8,773	2,007	10,780	34.4	8,703	1,633	10,336	8,528	1,221	9,749	6,924	653	7,577	24.2	38,442
		Qiantang	41,637	11,583	4,197	15,780	37.9	11,497	1,956	13,453	11,381	1,195	12,576	9,427	425	9,852	23.7	51,661
		Fengjing	752	351	8	359	47.7	348	5	353	341	3	344	279	0	279	37.1	1,335
	near central urban area	Xiaoshan	111,738	26,492	10,694	37,186	33.3	26,255	8,618	34,873	25,904	6,941	32,845	22,174	3,812	25,986	23.3	130,890
		Yuhang	76,113	22,650	9,618	32,268	42.4	22,449	7,624	30,073	22,182	6,182	28,364	19,659	3,243	22,902	30.1	113,607
		Linping	70,899	17,306	6,336	23,642	33.3	17,085	5,143	22,228	16,797	4,006	20,803	14,442	1,943	16,385	23.1	83,058
		Fuyang	40,883	10,010	3,653	13,663	33.4	9,994	2,932	12,926	9,795	2,303	12,098	8,425	1,330	9,755	23.9	48,442
		Linan	31,358	3,002	5,384	8,386	26.7	2,879	3,034	5,913	2,799	1,871	4,670	2,213	995	3,208	10.2	22,177
	remote areas	Tonglu	22,874	2,846	2,233	5,079	22.2	2,808	1,881	4,689	2,742	1,554	4,296	2,351	896	3,247	14.2	17,311
		Chunan	12,995	792	828	1,620	12.5	774	720	1,494	755	501	1,256	603	228	831	6.4	5,201
		Jiande	19,910	2,789	1,028	3,817	19.2	2,749	795	3,544	2,686	639	3,325	2,036	397	2,433	12.2	13,119
Gender		Male	337,832	96,217	30,099	126,316	37.4	94,969	22,208	117,177	93,334	16,821	110,155	78,966	8,878	87,844	26.0	441,492
		Female	312,117	88,671	27,699	116,370	37.3	87,536	20,511	108,047	86,159	15,627	101,786	73,203	8,183	81,386	26.1	407,589
Household registration		Local household registration	434,922	148,010	40,242	188,252	43.3	146,684	30,622	177,306	144,679	24,302	168,981	123,613	13,080	136,693	31.4	671,232
		non-local household registration	215,027	36,878	17,556	54,434	25.3	35,821	12,097	47,918	34,814	8,146	42,960	28,556	3,981	32,537	15.1	177,849
Year		2017	157,002	9,977	3,505	13,482	8.6	9,381	4	9,385	8,818	2	8,820	7,347	1	7,348	4.7	39,035
		2018	137,493	39,165	4,718	43,883	31.9	38,925	79	39,004	38,453	7	38,460	36,184	4	36,188	26.3	157,535
		2019	135,044	54,755	6,866	61,621	45.6	54,209	2,989	57,198	53,523	179	53,702	50,166	9	50,175	37.2	222,696
		2020	115,719	46,207	12,981	59,188	51.1	45,586	11,762	57,348	45,044	8,879	53,923	43,075	6,918	49,993	43.2	220,452
		2021	104,691	34,784	29,728	64,512	61.6	34,404	27,885	62,289	33,655	23,381	57,036	15,397	10,129	25,526	24.4	209,363
Total			649,949	184,888	57,798	242,686	37.3	182,505	42,719	225,224	179,493	32,448	211,941	152,169	17,061	169,230	26.0	849,081

### 3.2. The full course vaccination of PCV13

Among 649,949 children from 2017 to 2021, 169,230 were full course vaccinated with PCV13 according to the immunization procedure specified in the initial month of age, with an average vaccination rate of 26.0% (the fourth dose). From 2017 to 2021, 7,348, 36,188, 50,175, 49,993, and 25,526 children were full course vaccinated each year, with all course vaccination rates of 4.7, 26.3, 37.2, 43.2, and 24.4%, respectively. There is a statistically significant difference in the full course vaccination rate of PCV13 in the five years (*P* = 0.000), and there is an increasing trend (*P*_fortrend_ < 0.01). A total of 152,169 people were full course vaccinated with PCV13-CRM197 according to the immunization schedule specified for the initial month of age, with an average coverage rate of 23.4% (the fourth dose). A total of 17,061 people received full course PCV13-TT vaccine in accordance with the immunization schedule specified for the initial month of age, with an average coverage rate of 2.6% (the fourth dose). The full course vaccination rate of PCV13-CRM197 was higher than that of PCV13-TT. From 2017 to 2021, 7,347, 36,184, 50,166, 43,075, and 15,397 people were full course vaccinated with PCV13-CRM197, with vaccination rates of 4.7, 26.3, 37.2, 37.2, and 14.7%, respectively. There is a statistically significant difference in the full course vaccination rate of PCV13-CRM197 in the five years (*P* = 0.000). From 2017 to 2021, 1, 4, 9, 6,918, and 10,129 people were full course vaccinated with PCV13-TT, and the vaccination rates were 0, 0, 0, 6.0, and 9.7%, respectively. There was a statistically significant difference in the full course vaccination rate of PCV13-TT in the 5 years (*P* = 0.000) (Table 1).

### 3.3. The first vaccination of PCV13

From 2017 to 2021, 13,482, 43,883, 61,621, 59,188, and 64,512 people were vaccinated with the first dose, with vaccination rates of 8.6, 31.9, 45.6, 51.2, and 61.6%, respectively. The first vaccination rate of PCV13 in five years was different, the difference was statistically significant (*P* = 0.000), and there was an increasing trend (*P*_fortrend_ < 0.01). From 2017 to 2021, 9,977, 39,165, 54,755, 46,207 and 34,784 people were inoculated with the first dose of PCV13-CRM197, with vaccination rates of 6.4, 28.5, 40.6, 39.9, and 33.2%, respectively. The first vaccination rate of PCV13-CRM197 was different in 5 years, and the difference was statistically significant (*P* = 0.000). From 2017 to 2021, 3,505, 4,718, 6,866, 12,981, and 29,728 people were inoculated with the first dose of PCV13-TT, with vaccination rates of 2.2, 3.4, 5.1, 11.2, and 28.4%, respectively. The first vaccination rate of PCV13-CRM197 was different in 5 years, and the difference was statistically significant (*P* = 0.000) (Table 1). The distribution of first vaccination was different. Two months age group has the largest numbers of children being vaccinated with first dose, in contrast, children at 5 months of age is the smallest group inoculated with first dose. Among children born in 2017, the age group with the highest proportion of children who received the first vaccination was those aged over 6 months, accounting for 33.5% of overall vaccinated population in 2017. Among children born in 2021, the highest proportion of children who received the first vaccination at the age of ≤1 month is 40.8% (Table 2).

**Table 2:** Statistical table of the starting month age of the first dose of PCV 13 in Hangzhou from 2017 to 2021.

**Year**	**1 month^a^**			**2 month^b^**			**3 month^c^**			**4 month^d^**			**5 month^e^**			≥**6 month**			**Total**	
	**PCV13-CRM197**	**PCV13-TT**	≤ **1 month (proportion %)**	**PCV13-CRM197**	**PCV13-TT**	≤ **2 month (proportion %)**	**PCV13-CRM197**	**PCV13-TT**	≤ **3 month (proportion %)**	**PCV13-CRM197**	**PCV13-TT**	≤ **4 month (proportion %)**	**PCV13-CRM197**	**PCV13-TT**	≤ **5 month (proportion %)**	**PCV13-CRM197**	**PCV13-TT**	≥**6 month (proportion %)**	**PCV13-CRM197**	**PCV13-TT**
2017	443	0	443 (3.3)	2,457	0	2,457 (18.2)	2,806	0	2,806 (20.8)	2,387	1	2,388 (17.7)	872	1	873 (6.5)	1,012	3,503	4,515 (33.49)	9,977	3,505
2018	12,389	0	12,389 (28.2)	15,767	6	15,773 (35.9)	7,633	1	7,634 (17.4)	2,502	0	2,502 (5.7)	430	0	430 (1.0)	444	4,711	5,155 (11.75)	39,165	4,718
2019	21,984	2	21,986 (35.7)	19,889	0	19,889 (32.3)	9,639	6	9,645 (15.7)	2,725	1	2,726 (4.4)	322	2	324 (0.5)	196	6,855	7,051 (11.44)	54,755	6,866
2020	16,257	1,771	18,028 (30.5)	20,262	2,747	23,009 (38.9)	8,121	1,506	9,627 (16.3)	1,366	769	2,135 (3.6)	128	495	623 (1.1)	73	5,693	5,766 (9.74)	46,207	12,981
2021	16,116	10,170	26,286 (40.8)	13,812	10,134	23,946 (37.1)	4,229	4,501	8,730 (13.5)	575	1,754	2,329 (3.6)	33	911	944 (1.5)	19	2,258	2,277 (3.53)	34,784	29,728
Total	67,189	11,943	79,132 (32.6)	72,187	12,887	85,074 (35.1)	32,428	6,014	38,442 (15.8)	9,555	2,525	12,080 (5.0)	1,785	1,409	3,194 (1.3)	1,744	23,020	24,764 (10.20)	184,888	57,798

a 1 month refers to children who have received their first dose at the age of 6 weeks and < 2 months.

b 2 month refers to children who have received their first dose at the age of 2 months and < 3 months.

c 3 month refers to children who have received their first dose at the age of 3 months and < 4 months.

d 4 month refers to children who have received their first dose at the age of 4 months and < 5 months.

e 5 month refers to children who have received their first dose at the age of 5 months and < 6 months.

### 3.4. PCV13 vaccination of children in different regions

In the central urban area, 84,483 people were full course vaccinated, with a coverage rate of 32%. In the near central urban area, 78,236 people were full course vaccinated, with a coverage rate of 23.6%. In the remote area, 6,511 people were full course vaccinated, and the full course vaccination rate was 11.67%. There was a difference in the rate of the three regions, and the difference was statistically significant (*P* = 0.000). The full course vaccination rate was higher in the central urban areas than in near central urban areas, that in turn was higher than in remote areas (all *P*-values < 0.05). Among the children vaccinated with PCV13-CRM197, 80,266 people were full course vaccinated in the central urban area, with the vaccination rate of 30.5%. In total of 66,913 people were full course vaccinated in the near central urban area, with the vaccination rate of 20.2%. In the remote areas, 4,990 people were full course vaccinated, with the vaccination rate of 9.0%. There was a difference in the rate of the three regions, and the difference was statistically significant (*P* = 0.000). The full course vaccination rate was higher in the central urban areas than in near central urban areas, that in turn was higher than in remote areas (all *P*-values < 0.05). Among the children vaccinated with PCV13-TT, 4,217 people were full course vaccinated in the central urban area, with the vaccination rate of 1.6%. In total of 11,323 people were full course vaccinated in the near central urban area, with the vaccination rate of 3.4%. In the remote areas, 1,521 people were full course vaccinated, with the vaccination rate of 2.7%. There was difference in the rate of the three regions, and the difference was statistically significant (*P* = 0.000). The full course vaccination rate was higher in the near central urban areas than in remote areas, that in turn was higher than in central urban areas (all *P*-value < 0.05) (Table 1).

### 3.5. PCV13 vaccination of children in different genders

Among the 169,230 people who were full course vaccinated, 87,844 men were full course vaccinated, with a vaccination rate of 26.0%; 81,386 women were full course vaccinated, with a vaccination rate of 26.1%. There was no significant difference in vaccination rates between men and women (*P* = 0.502). Among the children who vaccinated PCV13-CRM197, 78,966 males and 73,203 females were full course vaccinated, with a vaccination rate of 23.4 and 23.5% respectively. There was no significant difference in vaccination rates between men and women (*P* = 0.450); Among the children who vaccinated PCV13-TT, 8,878 males and 8,183 females were full course vaccinated, with a vaccination rate of 2.6 and 2.6% respectively. There was no significant difference in vaccination rates between men and women (*P* = 0.877) (Table 1).

### 3.6. PCV13 vaccination of children in different household registration

Among 169,230 people who were full course vaccinated, 136,693 had local household registration, with vaccination rate of 31.4%, and 32,537 people had non-local household registration, with vaccination rate of 15.1%. The vaccination rates of the two household registration groups were different, and the difference was statistically significant (*P* = 0.000). Among the children who were vaccinated PCV13-CRM197, 123,613 people with local household registration and 28,556 with non-local household registration were full course vaccinated, with vaccination rate of 28.4 and 13.3% respectively. The vaccination rates of the two household registration groups were different, and the difference was statistically significant (*P* = 0.000). Among the children who were vaccinated PCV13-TT, 13,080 people with local household registration and 3,981 people with non-local household registration were full course vaccinated, with a vaccination rate of 3.0 and 1.9% respectively. The vaccination rates of the two household registration groups were statistically significantly different (*P* = 0.000) (Table 1).

## 4. Discussion

The main finding of this study is that the PCV13 vaccination rate was increasing year by year. The full course vaccination rate of PCV13 vaccine was different in different regions and different household registration population, but there was no difference by gender. The months at which the first dose of PCV13 was administered were scattered.

According to our analysis for 649,949 children aged no more than 6 years old born in Hangzhou in 2017–2021, the PCV13 vaccination rate in Hangzhou showed an increasing trend year by year, similar to that of Jinhua (33) and Tianjin (34). Although vaccination rate has increased from 31.9% in 2018 to 61.6% in 2021, higher coverage rates would be desirable to obtain a better group protection effect. PCV13 vaccination is also unbalanced among Hangzhou regions, and there is a large gap in vaccination rates among different cities. Although Hangzhou is located in economically developed eastern coastal area, there is still a large gap regarding the PCV13 vaccination rate compared with developed countries such as Europe and the United States. It was reported that 2 years after the introduction of PCV13 in the United States, the vaccination rate of PCV13 in children under 5 years old was 54% (35). In 2013, 64% of children under 2 years of age were vaccinated with more than one dose of PCV13 (36). In 2013, the vaccination rate of more than one dose of PCV13 in Argentina was 96%, and the vaccination rate of more than three doses was 81% (37). As a major developing country, India has also included PCV13 in India's national immunization program in stages in 2017/2018 (38). In China, the vaccination rate of PCV13 is far lower than the rates of vaccines included in national immunization program (39). This may be because in China, PCV13 has not been included in the national immunization program, and it belongs to the more expensive non-immunization program which is voluntarily vaccinated and paid by self. The low vaccination rate directly affects the establishment of the group protection effect of PCV13 in the population, and reduces the effect of population prevention of IPD. WHO recommends that all countries include pneumococcal polysaccharide conjugate vaccine (PCV) in their national immunization program, especially for those countries with a mortality rate of more than 50‰ of children under 5 years old which should make the introduction of multi-antigen PCV a high priority in their national immunization program (40). WHO also believes that improving the vaccination rate of PCV13 in children <2 years old can better prevent pneumonia and other diseases caused by Spn. The research of Ningbo University showed that in the case of herd immunity, when the price of each dose of vaccine is <740 yuan, the vaccination of PCV13 vaccine can save the direct cost while obtaining health benefits. When the price of each dose is between 740 yuan and 830 yuan, the PCV13 vaccine has a high cost effect; When the price of each dose is between 830 yuan and 1,100 yuan, the PCV13 vaccine is cost-effective; When the price per dose of vaccine is >1,100 yuan, the price per dose of vaccine is not cost-effective (32). The incremental cost-effectiveness ratio is 117,274 yuan, which is less than the per capita GDP of Ningbo City of 132,603 yuan in 2018, and much lower than the incremental cost of 812,419 yuan without considering herd immunity. There are also model studies to analyze the cost-effectiveness of PCV13 vaccination in Zhejiang Province (41). Therefore, the PCV13 volume-based procurement price control as well as the incorporation of PCV into the medical reimbursement list of Hangzhou are recommended by this study before the PCV13 was incorporated into national immunization scheme. It may be possible to encourage school-age children to vaccinate PCV13 by installment payment, to give certain financial subsidies to the vaccinated group, etc., in order to increase the vaccination rate, so as to reduce the occurrence of IPD in children.

In this study, the results showed that the full course vaccination of PCV13 in Hangzhou was different among different regions and household registration types. The overall coverage rate of PCV13 for children in the central urban areas was significantly higher than that in the remote areas, and the full course vaccination coverage rate of children with local household registration was significantly higher than that of children with non-local household registration, which was similar to the coverage rate of other non-immunization programs in China (42), and was consistent with the survey results of Quzhou (43) and Yinzhou in Ningbo (44), both in Zhejiang Province. Studies have shown that parents from more deprived families who do not pay enough attention to vaccines and have less access to vaccine-related information are generally less likely to take vaccination (45, 46). Furthermore, Hangzhou should carry out follow-up investigation of vaccination intention to find out the predictors related to vaccine hesitation. There is no difference in the full course vaccination rate between different genders, showing that there is no gender discrimination in PCV13 vaccination in Hangzhou. The PCV13 vaccination procedure is more flexible and complex than other multi-dose vaccination program. According to the situation of PCV13 vaccination for the first dose, the starting age of the first dose is relatively scattered, which means that many children cannot complete 4 doses of vaccination according to the procedure requirements. The later the start month, the fewer injections will be given. In general, most of the children in Hangzhou started their first vaccination within 2 months of age, which is consistent with the study in Ningbo (47). The proportion of children born in 2017 who received the first injection at the age of ≥6 months was the highest, and the starting age of the first injection was relatively late. This may be because PCV13-CRM197 had just been introduced into China <1 year, and some children were older children when they chose to receive the injection.

At the same time, this study also found that from 2017 to 2021, the vaccination rate of PCV13, whether the first injection, the full course vaccination rate, PCV13-CRM197 or PCV13-TT, is different and increasing (the all-process vaccination rate of the population born in 2021 is slightly lower than that of the previous year because some people do not reach the full course vaccination age). The first injection for children born in 2021 increased to the highest in the age group ≤1 month, indicating that parents of children at younger month pay attention to PCV13 and attach importance to the whole process of vaccination. This allows children to be vaccinated at an earlier age, which helps children develop immunity and get protection as early as possible.

The difference in the proportion of children vaccinated with PCV13-CRM97 and PCV13-TT in 2021 (14.71 vs. 9.68%) was the lowest in the series. This indicated that the proportion of children inoculated with PCV13-TT increased in children <2 years old. What causes parents' choice of PCV13 (PCV13-CRM97 or PCV13-TT) to change needs to be further investigated and verified.

This study has some limitations. Through the analysis of the existing vaccination data, although the vaccination differences among different populations with different characteristics were found, the reasons for the low full course vaccination rate of PCV13 in Hangzhou, the different vaccination rate in annual vaccination, the different vaccination rate in regions, the different months distribution of first dose vaccination and the different vaccination rates among different groups with registered residence were not investigated and analyzed. All the real causes affecting the inoculation of PCV13 in Hangzhou are not yet clear. At present, this study lacks data on the distribution of IPD/serotypes or clinical data (mortality rate, admission rate, etc.) from 2017 to 2021, making it impossible to analyze the protective effects after vaccination.

## 5. Conclusion

From 2017 to 2021, both the vaccination rate and the first dose vaccination rate of PCV13 for children under 6 years old in Hangzhou showed an increasing trend year by year, but the coverage of PCV13 for targeted population was still relatively low, and there were differences in vaccination among different regions and household registration.

## Data availability statement

The datasets presented in this study can be found in online repositories. The names of the repository/repositories and accession number(s) can be found in the article/Supplementary material.

## Author contributions

Conceived and designed the study: YL, XC, and QC. Obtained and organized the data: JuW, XC, YX, and WG. Analyzed the data: ZL, WJ, QX, and YY. Contributed reagents, materials, and analysis tools: JD, XiZ, XuZ, and JiW. Wrote the manuscript: XC and YL. English language modification: LG. All authors contributed to the article and approved the submitted version.
