# Supplementary material for: Analysis of 13-valent pneumococcal polysaccharide conjugate vaccine among children born in Hangzhou from 2017 to 2021
Source: Front Public Health. 2023 May 30;11:1184059. doi: 10.3389/fpubh.2023.1184059 (PMC10267870; doi:10.3389/fpubh.2023.1184059)
Supplement: Supplementary file 1 [file Table_1.DOCX]

Supplementary Material

Analysis of 13-valent pneumococcal polysaccharide conjugate vaccine among children born in Hangzhou from 2017 to 2021

Xinren Che^1^, Qinghua Chen^2^, Yan Liu^1^*, Lintao Gu^1^, Zhaojun Lu^1^, Wenwen Gu^1^, Jun Wang^1^, Wei Jiang^1^, Jian Du^1^, Xiaoping Zhang^1^, Yuyang Xu^1^, Xuechao Zhang^1^, Jing Wang^1^, Qixin Xie^1^, Yingying Yang^1^

^1^ Department of Expanded Program on Immunization, Hangzhou Center for Disease Control and Prevention, Hangzhou, Zhejiang, China

^2^ Department of Expanded Program on Immunization, Linping Center for Disease Control and Prevention, Linping District Hangzhou, Zhejiang, China

*** Correspondence:**Corresponding Author: Department of Expanded Program on Immunization, Hangzhou Center for Disease Control and Prevention, Hangzhou, 310021, Zhejiang, China

Corresponding Author: 250560301@qq.com

# Supplementary Data

**Sup1** χ2 value and *P* value

|  | | | No． of Children | Fitst dose | | | | | | | | | |  | Forth dose | | | | | | | | | |
| --- | --- | --- | --- | --- | --- | --- | --- | --- | --- | --- | --- | --- | --- | --- | --- | --- | --- | --- | --- | --- | --- | --- | --- | --- |
|  |  |  |  | PCV13-CRM197 | | | PCV13-TT | | | Total of the first dose | | | |  | PCV13-CRM197 | | | PCV13-TT | | | Total of the forth dose | | | |
|  |  |  |  | No． of vaccination | χ2 | *p* | No． of vaccination | χ2 | *p* | No． of vaccination | vaccination rates ofthe first dose（%） | χ2 | *p* |  | No． of vaccination | χ2 | *p* | No． of vaccination | χ2 | *p* | No． of vaccination | vaccination rates ofthe forth dose（%） | χ2 | *p* |
| Region | central urban areas | shagncheng | 75020 | 30526 |  |  | 4673 |  |  | 35199 | 46.9 |  |  |  | 24654 | 15766.245  8318.516,（ central urban, near central urban）  10916.113（central urban, remote areas）  4005.979（near central urban, remote areas） | ＜0.01 ＜0.05 ＜0.05 ＜0.05 | 788 | 1899.453 1903.725（central urban, near central urban ）  329.432（central urban, remote areas） 71.632（ near central urban, remote areas） | ＜0.01 ＜0.05 ＜0.05 ＜0.05 | 25442 | 33.9 | 11991.420 5281.616（central urban, near central urban ） 9419.611（central urban, remote areas）  3993.295（near central urban, remote areas） | ＜0.01 ＜0.05 ＜0.05 ＜0.05 |
|  |  | gongshu | 55937 | 24456 |  |  | 2388 |  |  | 26844 | 48.0 |  |  |  | 19586 |  |  | 754 |  |  | 20340 | 36.4 |  |  |
|  |  | xihu | 58469 | 23312 |  |  | 4751 |  |  | 28063 | 48.0 |  |  |  | 19396 |  |  | 1597 |  |  | 20993 | 35.9 |  |  |
|  |  | binjiang | 31364 | 8773 |  |  | 2007 |  |  | 10780 | 34.4 |  |  |  | 6924 |  |  | 653 |  |  | 7577 | 24.2 |  |  |
|  |  | qiantang | 41637 | 11583 |  |  | 4197 |  |  | 15780 | 37.9 |  |  |  | 9427 |  |  | 425 |  |  | 9852 | 23.7 |  |  |
|  |  | fengjing | 752 | 351 |  |  | 8 |  |  | 359 | 47.7 |  |  |  | 279 |  |  | 0 |  |  | 279 | 37.1 |  |  |
|  | near central urban area | xiaoshan | 111738 | 26492 |  |  | 10694 |  |  | 37186 | 33.3 |  |  |  | 22174 |  |  | 3812 |  |  | 25986 | 23.3 |  |  |
|  |  | yuhang | 76113 | 22650 |  |  | 9618 |  |  | 32268 | 42.4 |  |  |  | 19659 |  |  | 3243 |  |  | 22902 | 30.1 |  |  |
|  |  | linping | 70899 | 17306 |  |  | 6336 |  |  | 23642 | 33.3 |  |  |  | 14442 |  |  | 1943 |  |  | 16385 | 23.1 |  |  |
|  |  | fuyang | 40883 | 10010 |  |  | 3653 |  |  | 13663 | 33.4 |  |  |  | 8425 |  |  | 1330 |  |  | 9755 | 23.9 |  |  |
|  |  | linan | 31358 | 3002 |  |  | 5384 |  |  | 8386 | 26.7 |  |  |  | 2213 |  |  | 995 |  |  | 3208 | 10.2 |  |  |
|  | remote areas | tonglu | 22874 | 2846 |  |  | 2233 |  |  | 5079 | 22.2 |  |  |  | 2351 |  |  | 896 |  |  | 3247 | 14.2 |  |  |
|  |  | chunan | 12995 | 792 |  |  | 828 |  |  | 1620 | 12.5 |  |  |  | 603 |  |  | 228 |  |  | 831 | 6.4 |  |  |
|  |  | jiande | 19910 | 2789 |  |  | 1028 |  |  | 3817 | 19.2 |  |  |  | 2036 |  |  | 397 |  |  | 2433 | 12.2 |  |  |
| Gender |  | male | 337832 | 96217 |  |  | 30099 |  |  | 126316 | 37.4 |  |  |  | 78966 | 0.570 | 0.450 | 8878 | 0.240 | 0.877 | 87844 | 26.0 | 0.451 | 0.502 |
|  |  | female | 312117 | 88671 |  |  | 27699 |  |  | 116370 | 37.3 |  |  |  | 73203 |  |  | 8183 |  |  | 81386 | 26.1 |  |  |
| Household registration |  | Local household registration | 434922 | 148010 |  |  | 40242 |  |  | 188252 | 43.3 |  |  |  | 123613 | 18397.908 | ＜0.01 | 13080 | 752.313 | ＜0.01 | 136693 | 31.4 | 19845.818 | ＜0.01 |
|  |  | non-local household registration | 215027 | 36878 |  |  | 17556 |  |  | 54434 | 25.3 |  |  |  | 28556 |  |  | 3981 |  |  | 32537 | 15.1 |  |  |
| Year |  | 2017 | 157002 | 9977 | 56030.302 | ＜0.01 | 3505 | 65998.655 | ＜0.01 | 13482 | 8.6 | 96982.6 90748.76(for trend) | ＜0.01 ＜0.01 |  | 7347 | 62316.902 12090.49(for trend) | ＜0.01 ＜0.01 | 1 | 36999.203 28512.34(for trend) | ＜0.01 ＜0.01 | 7348 | 4.7 | 63711.443 28095.92(for trend) | ＜0.01 ＜0.01 |
|  |  | 2018 | 137493 | 39165 |  |  | 4718 |  |  | 43883 | 31.9 |  |  |  | 36184 |  |  | 4 |  |  | 36188 | 26.3 |  |  |
|  |  | 2019 | 135044 | 54755 |  |  | 6866 |  |  | 61621 | 45.6 |  |  |  | 50166 |  |  | 9 |  |  | 50175 | 37.2 |  |  |
|  |  | 2020 | 115719 | 46207 |  |  | 12981 |  |  | 59188 | 51.1 |  |  |  | 43075 |  |  | 6918 |  |  | 49993 | 43.2 |  |  |
|  |  | 2021 | 104691 | 34784 |  |  | 29728 |  |  | 64512 | 61.6 |  |  |  | 15397 |  |  | 10129 |  |  | 25526 | 24.4 |  |  |
